# Draft Genome Sequences of *Burkholderia contaminans* FFI-28, a Strain Isolated from a Contaminated Pharmaceutical Solution

**DOI:** 10.1128/genomeA.01177-16

**Published:** 2016-10-27

**Authors:** Maria Sol Haim, Marta Mollerach, Gary Van Domselaar, Sergio A. Teves, José Degrossi, Silvia T. Cardona

**Affiliations:** a Universidad de Buenos Aires, Facultad de Farmacia y Bioquímica, Cátedra de Microbiología, Buenos Aires, Argentina; b National Microbiology Laboratory, Public Health Agency of Canada, Winnipeg, Canada; c Universidad de Buenos Aires, Facultad de Farmacia y Bioquímica, Cátedra de Salud Pública e Higiene Ambiental, Buenos Aires, Argentina; d Department of Microbiology, University of Manitoba, Winnipeg, Canada; e Department of Medical Microbiology and Infectious Disease, University of Manitoba, Winnipeg, Canada

## Abstract

*Burkholderia contaminans* is a species of the *Burkholderia cepacia* complex, a group of bacteria that can grow in pharmaceutical products and are capable of infecting the immunocompromised and people with cystic fibrosis. Here, we report draft genome sequences for *Burkholderia contaminans* FFI-28, a strain isolated from a contaminated pharmaceutical solution.

## GENOME ANNOUNCEMENT

Microbial contamination of pharmaceutical products and personal care products (PPCPs) pose a health threat for people with diseases that compromise their immune system, including cystic fibrosis (CF) patients (1, 2). In North America, the most common objectionable microorganism recovered during PPCP recalls are *Burkholderia cepacia* complex (BCC) bacteria (2, 3), which are resistant to most antibiotics and cause infections in CF patients (4, 5).

*Burkholderia contaminans* (6) is an emerging species of the BCC in CF infections. *B. contaminans* is prevalent in Argentina (7) and Portugal (8) and is being increasingly isolated in CF patients from Spain (9) and Ireland (10). *B. contaminans* has been linked to outbreaks due to contaminated pharmaceutical products (11, 12). Genome sequences of *B. contaminans* isolated from an Argentinian CF patient (13) and from soil (14) have been reported; however, there are no genomic data of isolates obtained from PPCPs. Thus, genome sequence analysis is expected to shed light on whether *B. contaminans* has an enhanced capacity to survive in harsh environments and harbors genetic elements coding for virulence factors. Here, we used Illumina sequencing to obtain a draft genome of *B. contaminans* FFI-28, a strain isolated in Argentina from a pharmaceutical solution.

Cultures were grown in LB, and genomic DNA was isolated using phenol-chloroform as per Sambrook et al. (15). The isolated genomic DNA was converted into a sequencing-ready library using the Nextera XT DNA sample preparation kit (Illumina). Illumina sequencing was performed in a MiSeq personal sequencing system at the Next Generation Sequencing Core Platform at the Manitoba Institute for Children’s Health.

The average genome coverage was 34×. Sequenced reads were merged using FLASH version 1.3.0 (16) and assembled *de novo* using the SPAdes assembler version 1.2 (17). The assembly of FFI-28 generated 239 contigs greater than 1,000 bp with a mean contig length of 35,018 bp and an *N*_50_ of 72,119 bp. The predicted genome size was 8.37 Mb with a GC content of 66.3. Species in the genus *Burkholderia* are known for having large multipart genomes, and the sizes of our assemblies fell within the range of 7.4 to 9.73 Mb seen in previously sequenced genomes (18). Annotation of the assemblies with RAST (19) identified 7,744 open reading frames, which fall within the range previously seen in BCC genomes.

To classify *B. contaminans* FFI-28 according to multilocus sequence typing (MLST) (20), we retrieved the sequences of *recA*, *atpD*, *gltB*, *lepA*, *phaC*, *trpB*, and *gyrB* alleles and compared them with those of the BCC MLST website (http://pubmlst.org/bcc). The analysis classified *B. contaminans* FFI-28 as belonging to the globally distributed sequence type 102 (21). This genomic resource provides a starting point for elucidating the role of contaminated PPCPs as a source of infection with an emerging BCC pathogen.

### Accession number(s).

The *B. contaminans* FFI-28 draft genome has been deposited at DDBJ/ENA/GenBank under the accession number MDUI00000000. The version described in this paper is the first version, MDUI01000000.
